# Intrauterine device (IUD) migration to the fallopian tube: a rare location for a translocated IUD with no visceral injury

**DOI:** 10.1186/s40834-024-00278-8

**Published:** 2024-07-25

**Authors:** Peter Joseph Wangwe, Najma Awadh, Magreth Angelus

**Affiliations:** 1 Department of Obstetrics and Gynecology, Muhimbili University of Health and Allied Sciences, Dar es Salaam, Tanzania; 2 Department of Obstetrics and Gynecology, Shree Hindu Mandal Hospital, Dar es Salaam, Tanzania; 3 Department of Obstetrics and Gynecology, Muhimbili National Hospital, Dar es Salaam, Tanzania; 4 Shree Hindu Mandal Research and Training, Dar es Salaam, Tanzania

**Keywords:** Intrauterine devices, Migration, Silent uterine perforation, No visceral injury, Chronic pelvic pain

## Abstract

**Background:**

Loss of Intra Uterine Device (IUD) following silent perforation of the uterus either during or after IUD insertion is an uncommon finding due to a lack of immediate follow-up. We report a rare case in which uterine perforation following the migration of IUD to the right fallopian tube without visceral injury. The patient presented with lower abdominal pain and pain during sex for one year since IUD insertion. On examination, we noted tenderness on the right suprapubic region and on speculum examination, no IUD thread was seen. A radiological pelvic examination showed an empty uterus without an IUD. Laparotomy and retrieval of migrated IUD was done followed by repair of perforated uterus.

**Conclusion:**

Migrated IUD with silent uterine perforation without visceral injury is a distressing clinical condition both to the patient and the clinician. This case is reported to increase awareness in doing immediate vaginal examination and pelvic ultrasound post-IUD insertion.

## Introduction

Intrauterine devices (IUDs) are the most widely used form of long-acting reversible contraception because of their high efficacy, safety and low cost. However, in Tanzania the most commonly used modern method of family planning among women is implants (14%), followed by injectables (9%), IUD is not among the common methods used [12].

 Counselling during family planning service delivery is the key intervention in case there is a complication, and this is very well covered in Tanzania. Among the most common IUD-reported complications are uterine infection, expulsions, removals, and overall method discontinuation [3, 11]. These warrant close follow-up and immediate intervention where the need arises [8]. Post-insertion follow-up is needed and emphasis should be given to all clinicians to advise the clients for follow-up where speculum examination and pelvic ultrasound are done to assure the client following IUD insertion [7].

This is a case of silent uterine perforation presented with lower abdominal pain, and dyspareunia with no visceral perforation for one year. A case is presented to show how delay can lead to complications if timely intervention is not done.

## Case report

A 45-year-old female para 4 + 0 presented in the gynaecology clinic with complaints of lower abdominal pain and pain during sex for the past year since IUD insertion. The patient visited her Obstetrician about one year ago where an insertion of an IUD was done three months following normal vaginal delivery. Of note, the patient had a previous IUD device inserted four weeks postpartum, which was removed due to malposition. She gave a history of myomectomy before delivering the third baby.

**Fig. 1:**
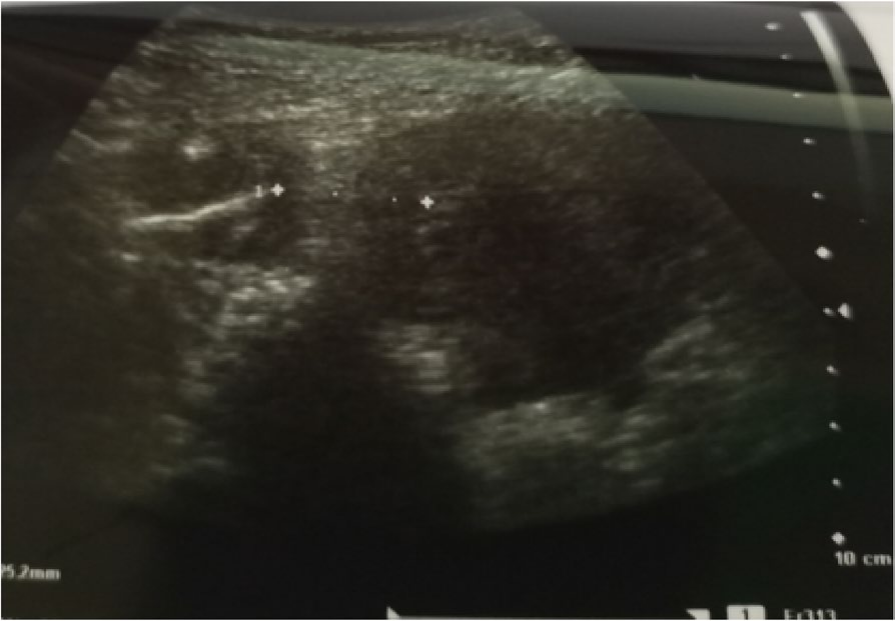
Abdominal USS Report

**Fig. 2:**
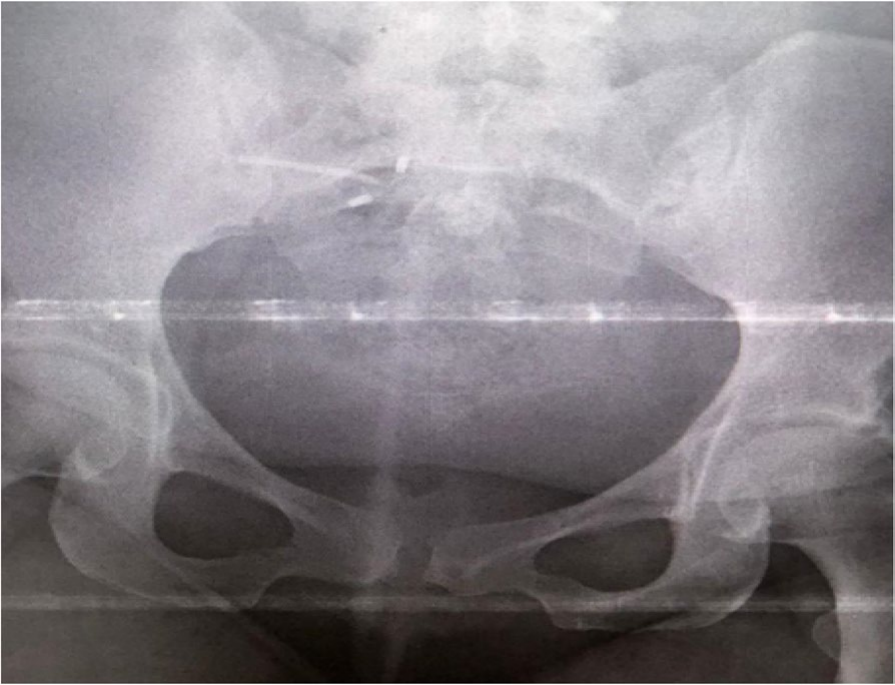
Plain Abdominal X ray

**Fig. 3:**
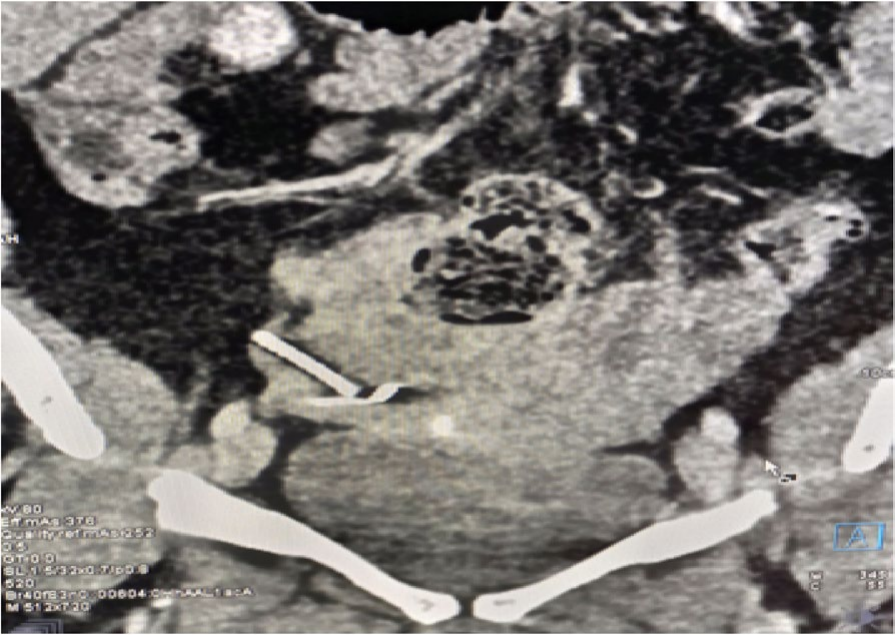
CT Scan of the Abdomen

**Fig. 4:**
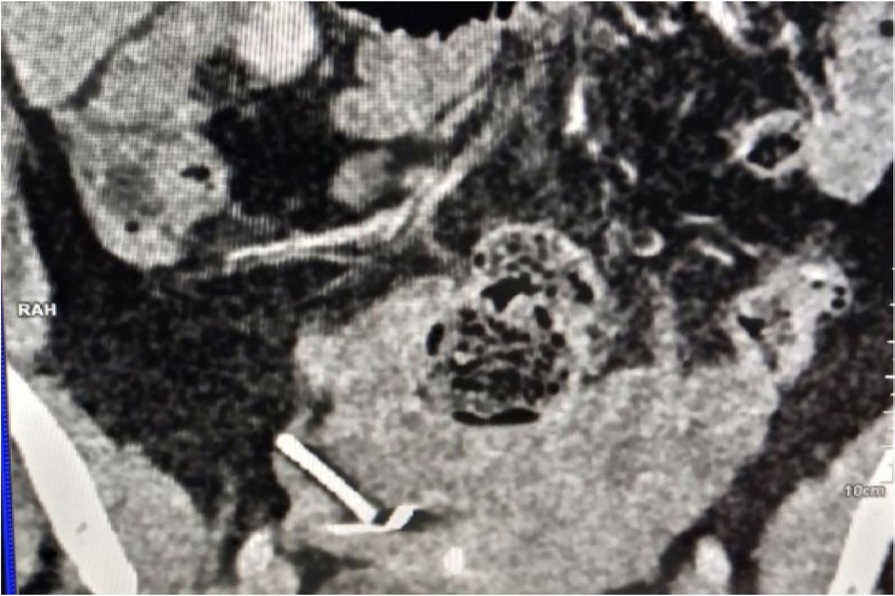
CT Scan of the Abdomen with Inverted T

She visited the same health facility with the above complaint and a gynaecological examination was done and the IUD string was not identified. The pelvic ultrasound was recommended, and the report revealed an IUD approximately 2.5 cm away from the fundus of the uterus to the right side and part of it to the peritoneum (Fig. 1). Transvaginal ultrasound examination did not visualize the IUD. An abdominal pelvic x-ray without contrast was recommended for further evaluation (Fig. 2) and demonstrated an IUD projecting into the pelvic region in an inverted T shape, thereby confirming that it had not been expelled. Confirmatory evaluation with computed tomography (CT) Figs. 3 and 4 reported displaced IUD in the right myometrium extending into the peritoneum.

**Fig. 5:**
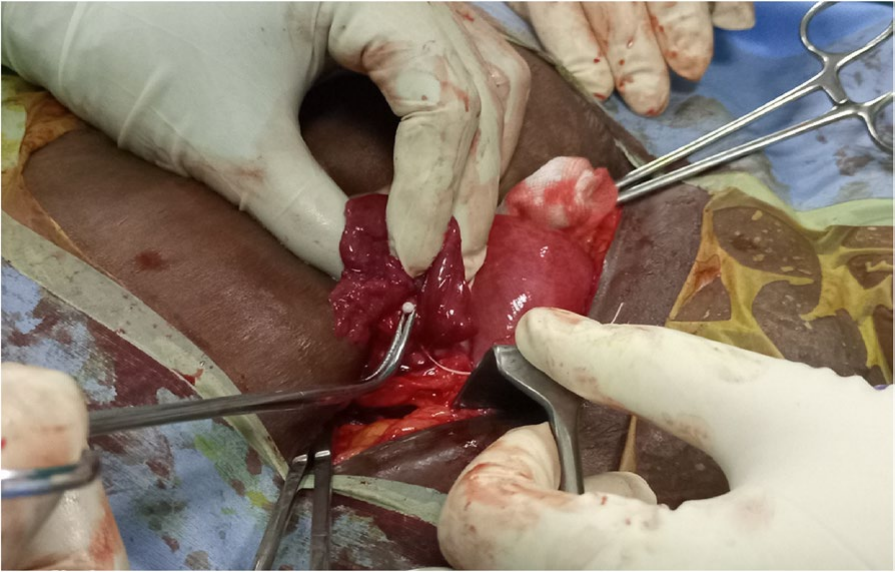
The tip of an inverted T shape of IUD in the parametrium

**Fig. 6:**
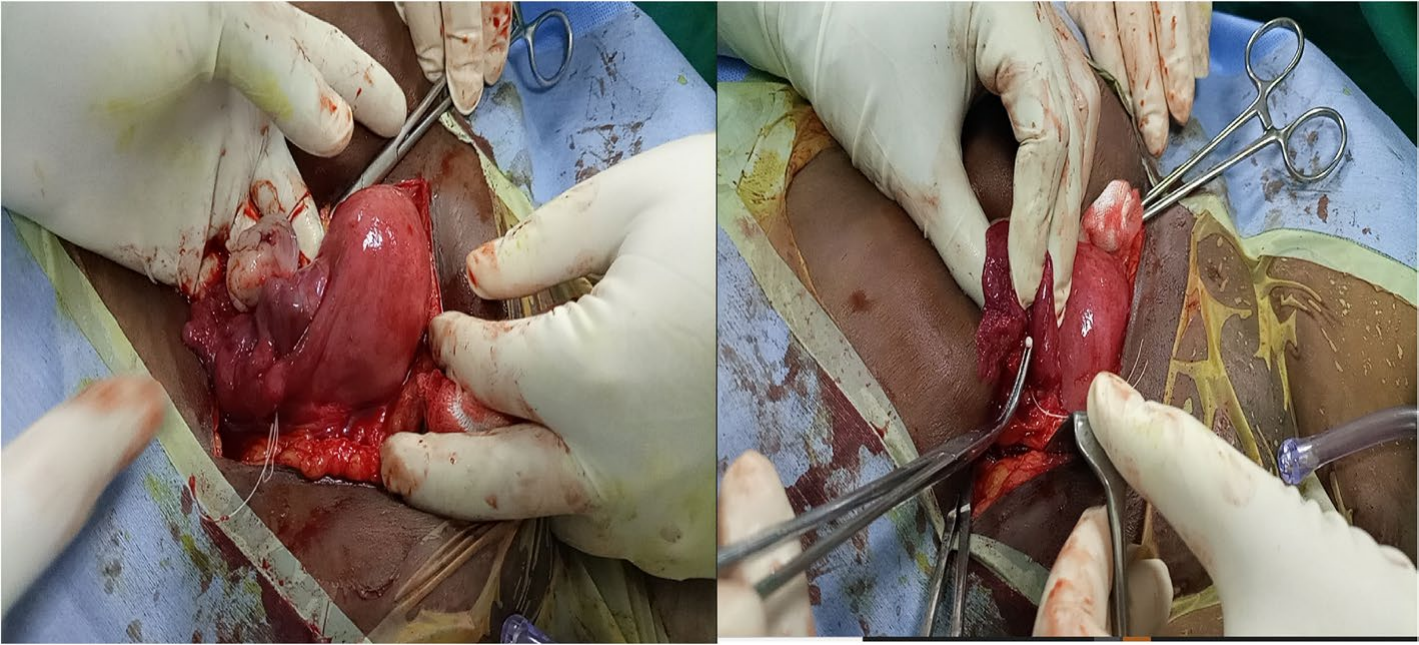
Health rt ovary, rt fallopian tube and uterus

**Fig. 7:**
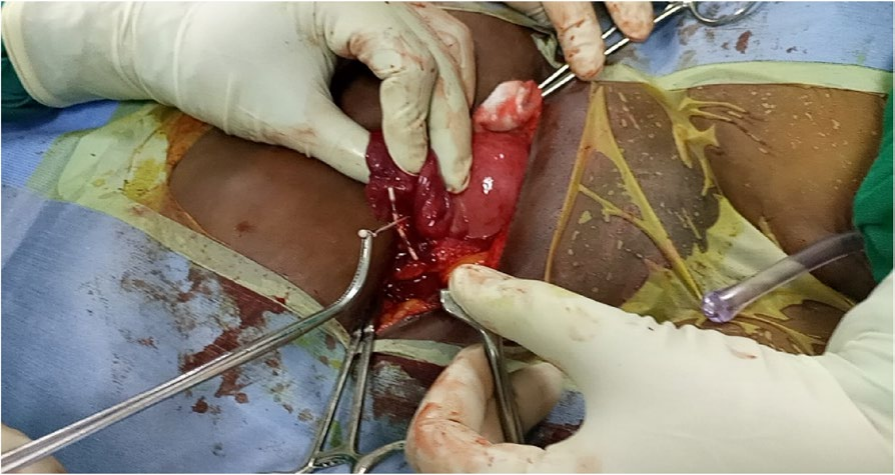
Process of extracting the IUD

**Fig. 8:**
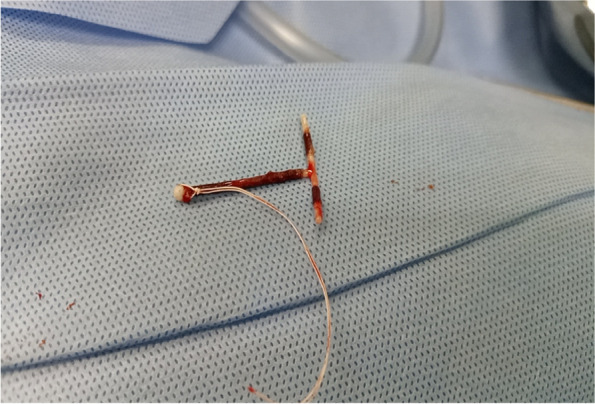
IUD after extraction

Blood chemistry and physical examination findings were within normal limits. The patient underwent a Hysteroscopic examination for IUD removal, which was not successful as there was no IUD in the uterine cavity. She was counselled for Laparotomy (Total Abdominal Hysterectomy) based on the previous Myomectomy and following the current incidence of lost IUD. However, the patient still needed more children and counselling for TAH was accepted with caution. Laparotomy was done and an IUD was identified in the right Fallopian tube with an inverted shape where the string was in the peritoneal cavity and the invited T shape in the parametrium (Fig. 5). The right fallopian tube and ovary were healthy (Fig. 6). The left fallopian tube and its adnexal were unremarkable. Extraction of the IUD was done through blunt dissection (Fig. 7), IUD extracted (Fig. 8) and there was no sign of infection detected. TAH differed as per the patient’s wishes for future fertility. The recovery from surgery was uneventful and the client opted for an alternative method of contraception.

## Discussion

The finding of IUD in the right fallopian tube with heath tube without sign of infection or adhesion as a result of inflammation is a rare condition. Despite of IUD being the most commonly reversible method used worldwide, some complications like uterine perforation are still reported [6, 11]. The risk factor for migration and uterine perforation varies depending on the patient’s factors like anatomical configuration of the uterus and adhesion due to previous uterine surgery [2]. Using a basic instrument designed for IUD insertion will minimize the uterine perforation by assessing the size, configuration, and uterine anomaly.

Most perforation occurs during insertion, and this is followed by immediate lower adnominal pain and pain during sex. This case report was typical where she presented with obvious risk for malposition, and pelvic pain for one year, yet the intervention was delayed. The clinician factor for perforation includes inadequate skills and counselling on what to do in case there is any complication.

In this case, the patient had a myomectomy which was followed by normal vaginal delivery. She also reported a history of IUD malposition, which poses a high suspicion index for complication and calls for close follow-up immediately after IUD insertion. Management of symptoms in most cases leads to delay in diagnosis of perforation and subsequently, unexpected complication in case visceral organs are involved [10]. Serial radiological investigation suffices in making a definitive diagnosis. It is documented that a plain pelvic X-ray is enough to make a definitive diagnosis [1] Fig. 2. However, in our case, several investigations were performed but all had the same finding warranting definitive treatment once perforation is suspected [5, 9].

The lost IUD is suspected immediately after the thread is not visualized during the speculum examination. Generally, abdominal pelvic ultrasound is the gold standard in confirming whether the IUD was expelled or migrated, and this will locate the site of the IUD. Any additional radiological investigation like plain abdominal x-ray, CT scan and MRI are ordered when there is suspected visceral injury. Although radiological report suggested location outside the uterine cavity, upon surgical exploration, the thread was lying in the peritoneal cavity and T shape was in the fallopian tube while another arm pointing to the myometrium just in the cornu of uterus which is a part of the fallopian tube, hence conclusion was termed to be in the fallopian tube. The serial radiological investigations has no added advantage rather increasing the cost of management of the patient as was observed in our client.

A minimally invasive procedure like Laparoscopy for a lost IUD is the gold standard of management. However, if there is suspected visceral perforation, Laparotomy will be indicated. The decision to perform laparotomy in this case was based on the factors such as potential for laparoscopy to fail, urgency of the situation and patient preference after being counseled on both options. The use of hysteroscopy for situations where ultrasound has confirmed migrated IUD has no place in patient management. Clinicians should always individualize patient management based on the investigation [4].

The future fertility after migrated IUD whether associated with visceral injury or not is something for discussion. Hysteroscopy which was done elsewhere reported a normal endometrial cavity. Findings from laparotomy were uneventful. However, the migrated IUD from the endometrial cavity to the parametrium and subsequently extraction through the abdomen pose some risk for infertility though it is beyond the scope of this case.

## Conclusion

Migrated IUD with uterine perforation without visceral injury is a distressing clinical condition. This case is reported to increase awareness and possibly prevent this avoidable uncommon complication by doing immediate vaginal examination and pelvic ultrasound post-IUD insertion.

## Data Availability

No datasets were generated or analysed during the current study.
